# Combined analysis of metabolome and transcriptome of wheat kernels reveals constitutive defense mechanism against maize weevils

**DOI:** 10.3389/fpls.2023.1147145

**Published:** 2023-05-09

**Authors:** Liangjie Lv, Xiaorui Guo, Aiju Zhao, Yuping Liu, Hui Li, Xiyong Chen

**Affiliations:** Crop Genetics and Breeding Laboratory of Hebei, Institute of Cereal and Oil Crops, Hebei Academy of Agriculture and Forestry Sciences, Shijiazhuang, China

**Keywords:** *Triticum aestivum* L., *Sitophilus zeamais*, metabolome, transcriptome, constitutive defense, flavonoids biosynthesis

## Abstract

*Sitophilus zeamais* (maize weevil) is one of the most destructive pests that seriously affects the quantity and quality of wheat (*Triticum aestivum* L.). However, little is known about the constitutive defense mechanism of wheat kernels against maize weevils. In this study, we obtained a highly resistant variety RIL-116 and a highly susceptible variety after two years of screening. The morphological observations and germination rates of wheat kernels after feeding ad libitum showed that the degree of infection in RIL-116 was far less than that in RIL-72. The combined analysis of metabolome and transcriptome of RIL-116 and RIL-72 wheat kernels revealed differentially accumulated metabolites were mainly enriched in flavonoids biosynthesis-related pathway, followed by glyoxylate and dicarboxylate metabolism, and benzoxazinoid biosynthesis. Several flavonoids metabolites were significantly up-accumulated in resistant variety RIL-116. In addition, the expression of structural genes and transcription factors (TFs) related to flavonoids biosynthesis were up-regulated to varying degrees in RIL-116 than RIL-72. Taken together, these results indicated that the biosynthesis and accumulation of flavonoids contributes the most to wheat kernels defense against maize weevils. This study not only provides insights into the constitutive defense mechanism of wheat kernels against maize weevils, but may also play an important role in the breeding of resistant varieties.

## 1 Introduction

Wheat is an important crop for human food and livestock feed; it provides essential nutrients for human diet (Shewry, 2009). Wheat grains are seasonal, so almost all harvest kernels must be stored for continuous consumption. Maize weevil (*Sitophilus zeamais*), one of the most destructive insect pests of stored grains, infects grains in the field and during the storage period, resulting in a serious loss in wheat quantity and quality (Vásquez-Castro et al., 2012; Keskin and Ozkaya, 2014). Maize weevils and other stored grain pests not only devour the whole grain but also cause heat, mildew infection, and caking of stored grain, which significantly decrease grain quantity and compromise the safety of grain storage (Edde, 2012; Antunes et al., 2016). After the kernels are infected by stored grain pests, the fat content and gluten content, as well as, sedimentation value decrease.Additionally the acidity increases, that adversely affect the quality of wheat storage and processing (Keskin and Ozkaya, 2014; Nietupski et al., 2021). In addition, stored pests excrete feces and larval fragments also affecting quality of the infested grain (Ortegadorame, 1997; Edde, 2012). The quantity and quality of stored wheat grains are directly related to the quality of human life and food safety. Thus, the management of the stored grain pests, especially maize weevils has been an important concern and research interest.

Traditional methods to control maize weevils included fumigants (e.g., phosphine, methyl bromide, Korean spices, and medicinal plants) and insecticides (e.g., organophosphate fenitrothion, pyrethroid, and deltamethrin) (Lee et al., 2001; Rajendran and Gunasekaran, 2002; Muhareb, 2010; Vásquez-Castro et al., 2012; Haddi et al., 2018; Singh et al., 2021). However, the recurrent use of fumigants and insecticides lead to marked increase of genetic resistance in stored insect pests (Haddi et al., 2018; Nayak et al., 2019; Singh et al., 2021). In addition, methyl bromide depletes the ozone in the atmosphere and several insecticides leave behind hazardous residues in stored grain, which seriously threaten environmental safety, food quality, and human health (Sgarbiero et al., 2003; Joia et al., 2006; Phillips and Throne, 2010; Haddi et al., 2018). The aforementioned limitations of chemical methods motivated research into various alternatives to control stored insects. Improving the defensive ability of host plants by breeding is the most sustainable, effective and eco-friendly method. With long period of evolution, plants developed various defense strategies to cope with insect attacking, which are summarized as constitutive defense and induced defense (Wu and Baldwin, 2010; Schuman and Baldwin, 2016). Constitutive defense is produced irrespective of whether insects are present, including physical barriers (the formation of thorns, trichomes, and cuticles) and chemical barriers (the formation of defensive metabolites or proteins in plant special tissues, such as flavonoids, terpenoids, and alkaloids) (Howe and Jander, 2008; Wu and Baldwin, 2010; War et al., 2012). The induced defense of host plants is activated following infestation by insects. When attacked by insects, host plants recognize and transmit insect feeding signals by a series of networks, which activate the expression of defense-related genes to synthesize the compounds associated with insect resistance in host plants (Wu and Baldwin, 2010; War et al., 2012; Aljbory and Chen, 2018). Therefore, understanding the defense mechanisms and effectively utilizing the defense-related genes and metabolites are necessary to enhance wheat resistance to maize weevils.

The defense mechanisms of plants are extremely complex and involved the interaction between genes and metabolites. A single metabolome or transcriptome cannot systematically explain the defense mechanism. The combined transcriptomic and metabolomic analysis reveal the differential transcript and metabolite levels along with their relationships (Liu et al., 2022). This technique provided a powerful strategy to directly and comprehensively elucidate the defense mechanisms of host plants to insects and was widely utilized in recent years. For examples, the combined transcriptome and metabolome analysis of rice attacked by the Asian rice gall midge indicated that rice released reactive oxygen species which led to insect mortality by limiting nutrient supply (Agarrwal et al., 2016). The transcriptomic and metabolomic data showed that cotton synthesized methyl salicylate to attract the parasitoid *Peristenus spretus* which led to indirect plant defense against *Apolygus lucorum* infection (Huang et al., 2021). Additionally, the integrated analysis of transcriptome and metabolome was used to elucidate the maize response to *Ostrinia furnacalis* feeding, which found that the defense of maize was mediated by phytohormones, benzoxazinoids, and volatiles (Guo et al., 2019). In wheat, Wang et al. used a combined transcriptome and metabolome analysis to study the defense mechanism following the *Sitodiplosis mosellana* attacking, which showed that phenylalanine and flavonoid pathway play influential roles in wheat grain defense (Wang et al., 2022). However, previous studies were mainly focus on induced defense mechanism, studies related to constitutive defense mechanisms of host plants to insects were rarely reported. Thus, we investigated the constitutive defense mechanism of wheat kernels to maize weevils using the combined transcriptomic and metabolomic approach.

In this study, the transcriptome and metabolome were analyzed to determine the differences in gene expression and metabolite accumulation between the resistant variety RIL-116 and the susceptible variety RIL-72, as well as identified the key metabolic pathways related to defense against maize weevils. We further constructed the metabolic regulatory network and determined several key genes and metabolites contributing to the constitutive defense of wheat kernels *via* combined transcriptomic and metabolomic analysis. This study not only provide a more comprehensive understanding of the constitutive defense mechanism in wheat kernels but also established the theoretical basis for the utilization of defense-related genes and metabolites.

## 2 Materials and methods

### 2.1 Wheat resistance screening and maize weevil feeding treatments

A total of 198 self-constructed recombinant inbred lines (RIL, the 7th generation) using Zhoumai 16 and Gaoyou 8901 as parents were planted within the experimental plot at the Institute of Cereal and Oil Crops, Hebei Academy of Agriculture and Forestry Sciences (HAAFS), Gaocheng District (37°95′N, 114°71′E), Shijiazhuang City, Hebei, China, during the 2017-2018 and 2018-2019 growing seasons. Each variety was planted with three replications in a row 2.0 m long with 25 cm spacing between the rows. An average of 40 seeds were sown in each row. All fields were managed normally without pesticides. After maturity, the wheat kernels were harvested and stored at a granary in an experimental station with approximately 13% moisture content. A temperate monsoon climate is characteristic of the study area, with temperatures after wheat harvest (June to September) averaging 26.30°C, average humidity of 76.67%, and a mean rainfall of 115 millimeters (https://www.ncei.noaa.gov/). The maize weevils used in this study were from stored wheat in Institute of Cereal and Oil Crops, Hebei Academy of Agriculture and Forestry Sciences, and were kept in culture on whole wheat kernels in incubator with 27°C, 75% RH. Concerning the maize weevil treatment, three replicates of each harvest variety were mixed and transferred into an incubator with adult maize weevils which were two weeks after emergence for 50 days. The maize weevils were allowed to freely gnaw, and the highest resistant and susceptible varieties were identified.

### 2.2 Morphological observations and germination rate statistics

After two years of screening, the resistant variety RIL-116 and susceptible variety RIL-72 were obtained and planted with three replicates in the 2019-2020 growing season. Each replicate containing approximately 40 seeds were planted in a single row with a length of 2.0 m and row spacing of 25 cm. After maturity, the wheat kernels of RIL-116 and RIL-72 were harvested and followed by maize weevils freely feeding treatment. 100 RIL-116 and RIL-72 wheat grains were randomly selected and placed on culture plates to record the morphological characteristics. The seeds were germinated in the illumination germination box, and the germination rate was calculated. The morphological characteristics of eroded grains and germinated seeds were imaged with a digital camera (Cannon90D).

### 2.3 Metabolome analysis

At the grain filling stage on day 35, a total of six group, including three replicates of susceptible variety RIL-72 which was designated as CK (CK1, CK2, and CK3) and three replicates of resistant variety samples RIL-116 (IR1, IR2, and IR3), were collected and analyzed by metabolomics profiling. Biological samples were freeze-dried by vacuum freeze-dryer (Scientz-100F) for metabolome analysis. The metabolome sequencing was performed by Wuhan Metware Biotechnology Co., Ltd (Wuhan, China). The freeze-dried samples were crushed using a mixer mill (MM 400, Retsch) with a zirconia bead for 1.5 min at 30 Hz. A 100 mg of lyophilized powder was dissolved in 1.2 mL 70% methanol solution, vortexed for 30 seconds every 30 minutes for 6 times in total. The samples were placed in a refrigerator at 4°C overnight. Following centrifugation at 12000 rpm for 10 min, the extracts were filtreted (SCAA-104, 0.22 μm pore size; ANPEL, Shanghai, China, http://www.anpel.com.cn/) before UPLC-MS/MS analysis. A 75μL centrifuged supernatant was transferred to a fresh glass vial for UPLC-MS/MS analysis. The quality control (QC) and qualitative and quantitative analysis of metabolites were performed as described by of Lv et al. (Lv et al., 2022). The principal component analysis (PCA) of all samples was completed using R software (http://www.r-project.org/). The differential metabolites were selected based on the combination of a statistically significant threshold of variable influence on projection (VIP) values obtained from the OPLS-DA model and fold change from the ratio of resistant plants (IR) to susceptible plants (CK). Metabolites with VIP ≥ 1.0 and fold change ≥ 2 or fold change ≤ 0.05 were considered as differential metabolites. Hierarchical clustering (Euclidean distance) was performed with MeV4.9 to explore the pattern of metabolite abundance. The differential metabolites were annotated using the Kyoto Encyclopedia of Genes and Genomes (KEGG) database, followed by enrichment pathway analysis.

### 2.4 Transcriptome analysis

The grain filling stage of wheat is appromately five weeks. At the grain filling stage, three biological replicates of kernels from resistant and susceptible varieties were collected on days 7, 14, 21, 27 and 35. A total of 100 kernels from 25 plants were collected for each replicate. A total of 30 individual samples were used for RNA-Seq analysis. The transcriptome sequencing was performed by Wuhan Metware Biotechnology Co., Ltd (Wuhan, China). The total RNA was extracted using TRIzol reagents from Invitrogen (CA, USA). Based on the instructions provided by the manufacturer (Illumina), 100ng RNA was used to construct the RNA-seq library and cDNA was synthesized with SuperScript II reverse transcriptase (Invitrogen, CA, USA). After the second-strand cDNA was synthesized and linked, The cDNA was purified by AMPure XP system and the library quality was assessed on the Agilent Bioanalyzer 2100 system. Then, the enriched and purified cDNA fragment was sequenced on the Illumina HiSeq platform. In order to generate clean readings, low-quality raw readings were filtered out of sequenced raw readings. The clean reads were mapped to wheat reference genome (http://plants.ensembl.org/Triticum_aestivum/Info/Index) to get location information using HISAT242. Differentially expressed genes (DEGs) of resistant plants relative to susceptible plants were detected with a threshold of |log_2_FC| ≥ 1 and a false discovery rate (FDR)<0.05. The DEGs were annotated by the Gene ontology (GO) and Kyoto Encyclopedia of Genes and Genomes (KEGG) databases. GO categories and KEGG pathway enrichment of DEGs were generated using R software. The RNA-seq reads were available at the NCBI Sequence Read Archive (https://www.ncbi.nlm.nih.gov/bioproject/PRJNA803964).

### 2.5 Weighted gene co-expression network analysis

The WGCNA was performed using the WGCNA package in R. After the filtration of low expression genes with FPKM < 0.1, the expression values of DEGs were imported into WGCNA package to construct co-expression modules. The soft threshold power in this study was set as 15. The co-expression modules were construct using the WGCNA algorithm and visualized using the WGCNA dendrogram and color function. The dynamic tree cut method, which merged highly correlated modules using a correlation coefficient greater than 0.75, was used to further determine the co-expression gene modules of the gene dendrogram. To identify the important modules, the association between modules and samples were calculated using the default settings and visualized by a heat map. The Connectivity between genes was calculated, and the genes with high connectivity (K value) in each module were considered as hub genes. Finally, the hub genes and their highly connected genes in specific modules were identified and visualized by Cytoscape software.

### 2.6 Correlation analysis of transcriptomic and metabolomic data

We determined the correlation between DEGs and differenrially accumulated metabolites (DAMs), and utilized the cor function in R to calculate the Pearson correlation coefficient and p values. The correlation coefficients between genes and metabolites greater than 0.8 were selected to draw a correlation cluster heat map. In order to identify the common pathways, DEGs and DAMs were simultaneously mapped to the KEGG database. Using a coefficient method, the correlation network diagram between genes and metabolites from common KEGG pathways were visualized using Cytoscape software. To further elucidate the relationship between genes and corresponding metabolites, the KEGG pathway map was constructed.

## 3 Results

### 3.1 Evaluation of resistance and morphological characteristics of wheat varieties to maize weevils

**Figure 1:**
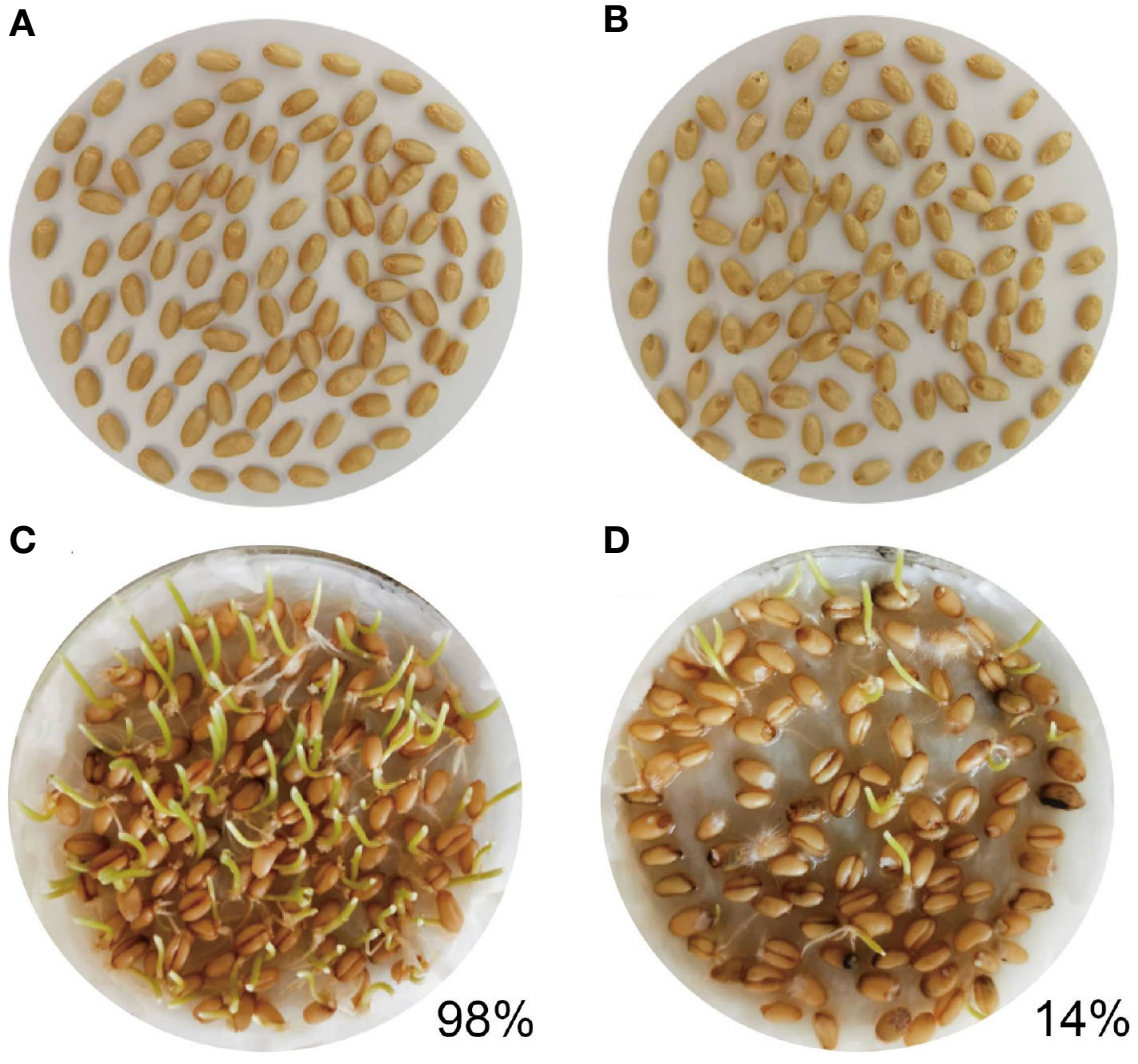
The characteristics and germination test of RIL-116 and RIL-72 after maize weevils attacking. **(A, B)** The characteristics of RIL-116 and RIL-72 after maize weevils attacking: **(A)** RIL-116, **(B)** RIL-72. **(C, D)** The germination test of RIL-116 and RIL-72 after maize weevils attacking: **(C)** RIL-116, **(D)** RIL-72.

The resistance of the tested wheat RIL varieties against maize weevil was observed and calculated (**Figure S1**). The RIL-116 variety was found to be highly resistant to maize weevils after two years of screening, while the RIL-72 was susceptible to maize weevils. Two other varieties, RIL-119 and RIL-13, showed middle levels of resistance to the maize weevils during two consecutive years of growing season. In 2019-2020, the RIL-116 and RIL-72 varieties were mainly planted and harvested. Three repeated mixtures of each experimental material were used to observe morphology. The kernels of RIL-116 and RIL-72 were stored in the same environment with maize weevils freely feeding, which resulted in infection of most of RIL-72 wheat grains by maize weevils and exhibited obvious brown and decayed. Under prolonged of infection, wheat kernels fed by maize weevils gradually became empty (**Figure 1A**). By contrast, only few RIL-116 kernels were attacked by maize weevils and turned slightly brown (**Figure 1B**). Additionally, the germination test was performed to assess the viability of kernels. The results showed that the germinate rate of RIL-116 (closed to 98%) was significantly higher than that of RIL-72 (only 14%) (**Figures 1C, D**). The results indicated that maize weevils preferred to feed on susceptible variety RIL-72 but not the resistant variety RIL-116. Thus, we conjectured that RIL-116 likely contained several special substances, which contributed to greater defense ability against maize weevils.

### 3.2 Metabolite profiles of RIL-72 and RIL-116

To clarify the endogenous defense mechanism of wheat against maize weevils, three biological replicates of each RIL-72 and RIL-116 wheat kernels at 35days after grain filing stage were used for metabolome analysis by UPLC-MS/MS. Based on the qualitative and quantitative analysis, a total of 509 metabolites were identified in six wheat kernels samples including 3 terpenoids, 2 tannins, 64 phenolic acids, 63 others, 51 organic acids, 41 nucleotides and derivatives, 67 lipids, 9 lignans and coumarins, 87 flavonoids, 75 amino acids and derivatives, and 47 alkaloids (**Figure S2A** and **Table S1**). The PCA was used to understand the overall differences of metabolic profile between inner and inter-group variations of RIL-72 (CK) and RIL-116 (IR). The two-dimensional PCA plot revealed three biological replicates of each variety clustered together, indicating high repeatability among same varieties. Whereas, the metabolic profiles between two varieties presented separation trend, which certified significant differences in these metabolites between inter-group. (**Figure S2B**). Additionally, OPLS-DA was performed to facilitate the identification of DAMs. The Q^2^ > 0.9 and P< 0.5 of OPLS-DA verification diagram indicated the robustness and reliability of the model (**Figure S2C**). Thus, the VIP of OPLS-DA model combined with fold change was used to screen DAMs.

**Figure 2:**
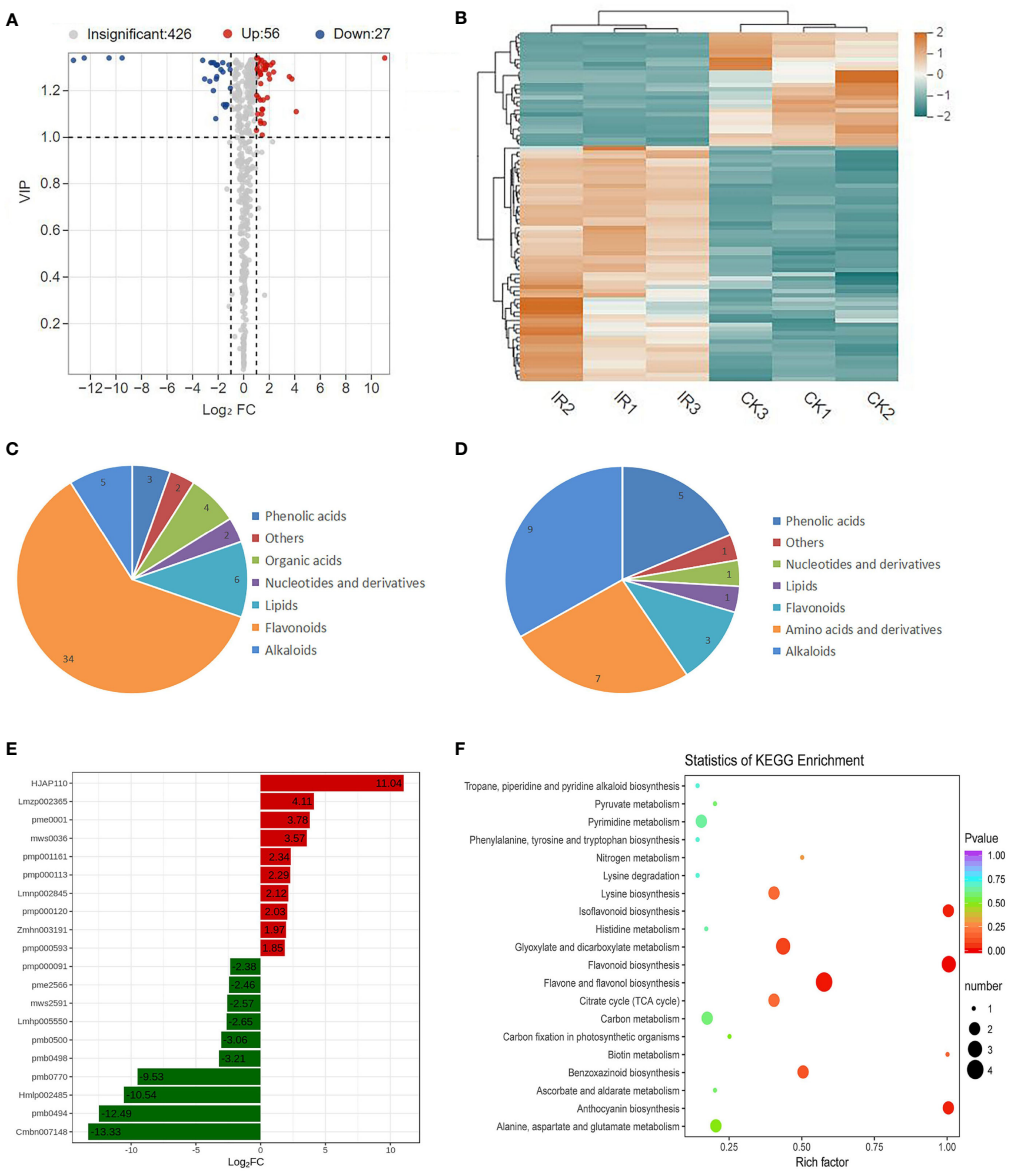
The metabolic profiling of RIL-116 and RIL-72. **(A)** Volcano plot of differential metabolites in the comparison of RIL-116 (IR) and RIL-72 (CK). Each point in the volcano map represents a metabolite, red represent up-accumulated metabolites and blue represent down-accumulated metabolites. **(B)** Clustering heat map of differential metabolites. The level of each metabolites relative content were depicted by color range from blue (low) to red (high). **(C)** The number of classification of up-accumulated metabolites. **(D)** The number and classification of down-accumulated metabolites. **(E)** The abundance histogram of top 10 up-accumulated metabolites (red-bars) and top 10 down-accumulated metabolites (green bars). **(F)** KEGG analysis of differentially accumulated metabolites.

Based on a VIP ≥ 1.0 and a fold change ≥ 2 or ≤ 0.05, a total of 83 DAMs were obtained in IR vs. CK, including 56 up-accumulated and 27 down-accumulated metabolites (**Figure 2A** and **Table S1**). The differential metabolites were visualized using the heatmap with hierarchical cluster analysis shown in **Figure 2B**, which further demonstrated the significant difference in metabolites between IR and CK. These DAMs might be related to the difference resistance between resistant and susceptible wheat varieties. The 56 up-accumulated metabolites were divided into seven classes, mainly including flavonoids, lipids, and alkaloids. Among the up-accumulated metabolites, flavonoids accounted for the largest proportion (**Figure 2C**). The main down-accumulated metabolites were alkaloids, amino acids, and andphenlic acids (**Figure 2D**). To further find the defense-related metabolites, the top 10 up-accumulated and down-accumulated DAMs were screened and the detailed information were listed in **Figure 2E** and **Table S2**. Compared with CK, myricetin-O-rhamnoside was the most up-accumulated metabolite with the highest log2 fold change value of 11.04. 1-O-Feruloyl-3-O-caffeoylglycerol showed the lowest log2 fold change value of -13.33 and thus was the most down-accumulated metabolites. Flavonoids were reported contained 10 major subgroups: chalcones, aurones, flavanones, flavones, isoflavones, dihydroflavonols, flavonols, leucoanthocyanidins, anthocyanidins, and flavan-3-ols (Nakayama et al., 2019). Notably, all of the top 10 up-accumulated metabolites were flavonoids, including 5 flavonoid, 2 flavonols, 1 flavonoid carbonoside, and 2 dihydroflavonol (**Table S2**). Thus, we speculated that accumulation of flavonoids was the most highly related to resistance of wheat to maize weevils.

In order to explore the key metabolic pathway involving in wheat defense against maize weevils, the KEGG annotation and enrichment analysis of DAMs were performed. A number of KEGG pathways were enriched and the top 20 pathways were listed in **Figure 2F**. KEGG pathway analysis showed that the DAMs were mainly enriched in the following six pathways: flavonoid biosynthesis, flavone and flavonol biosynthesis, isoflavonoid biosynthesis, anthocyanin biosynthesis, glyoxylate and dicarboxylate metabolism, and benzoxazinoid biosynthesis, which indicated that these pathways might be involved in wheat defense against maize weevils. Among the above pathways, flavonoid biosynthesis, flavone and flavonol biosynthesis, isoflavonoid biosynthesis, and anthocyanin biosynthesis were the branches of flavonoids biosynthesis. This result indicated that flavonoids biosynthesis-related pathway was the most key metabolic pathway related to wheat defense against maize weevils. Taken together, the metabolic results suggested that the biosynthesis and accumulation of flavonoids contribute the most to wheat defense against maize weevils.

### 3.3 Transcriptome analysis of RIL-72 and RIL-116

To explore the dynamic molecular events of metabolic differences at the transcriptional level, the wheat kernels of RIL-72 (CK1-5) and RIL-116 (IR1-5) at five different grain filling stages were collected for transcriptome analysis. The numbers of 1, 2, 3, 4, and 5 represented days 7, 14, 21, 27, and 35 after grain filling stage, respectively. After removing low quality reads, a total of 420.87 Gb of clean bases were generated from 30 samples (three biological replicates for each stage). Each sample contained at least 12 Gb with GC percentages of 53.61%-60.27%. The quality scores of Q20 were 96.99%-97.93% and those of Q30 were 92.34%-94.1% (**Table S3**). When the clean data were mapped to the wheat reference genome, the proportion of clean reads successfully matching to the wheat genome was higher than 85.74% (**Table S3**). Overall, these results indicated the high quality of transcriptome data for further analysis.

**Figure 3:**
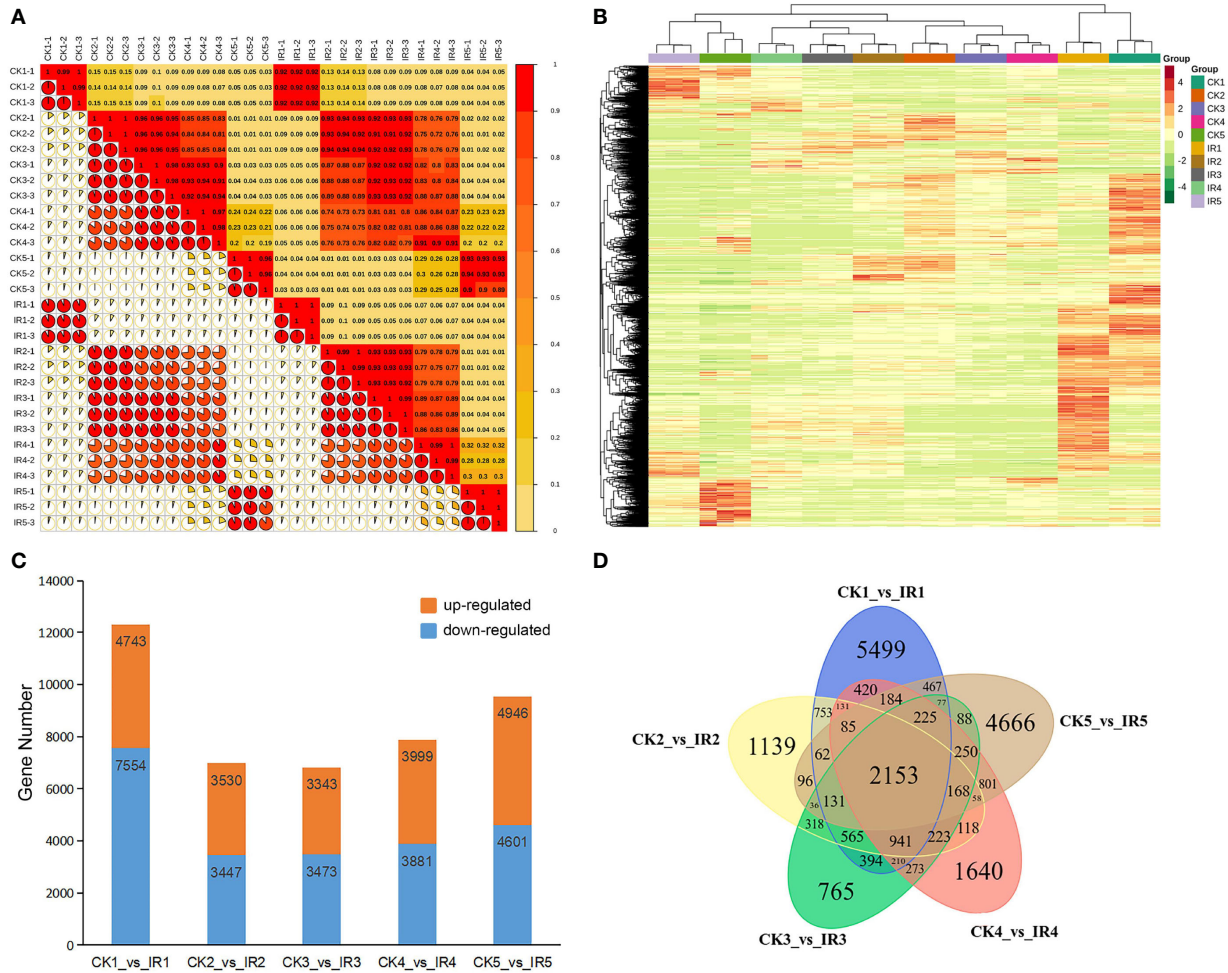
The overall analysis of RIL-116 and RIL-72 transcriptomic data. **(A)** Correlation analysis of all samples. **(B)** Clustering heat map of all DEGs from 30 samples. The relative expression level of each genes depicted by color range from green (low) to red (high). **(C)** The number of up- and down-regulated DEGs in five comparisons (IR1 vs. CK1, IR2 vs. CK2, IR3 vs. CK3, IR4 vs. CK4, and IR5 vs. CK5). **(D)** The venn diagram of DEGs in five comparisons to display the common and unique number of DEG.

Pearson’s correlation coefficient (r) was used as an index to evaluate biological repeated correlation. R^2^> 0.8 between three biological repeats of each wheat group suggested good stability and reproducibility of transcriptome data in the tested wheat kernels and the high reliability of the experimental methods (**Figure 3A**). Further, the DEGs were identified according to |log2 fold change| ≥ 1 and FDR< 0.5. The hierarchical clustering heatmap of total DEGs obtained from all samples was presented in **Figure 3B**, which showed distinct expression level of DEGs among different samples. A total of 12297 DEGs (4743 up-regulated and 7554 down-regulated), 6977 DEGs (3530 up-regulated and 3447 down-regulated), 6817 DEGs (3343 up-regulated and 3474 down-regulated), 7880 DEGs (3999 up-regulated and 3881 down-regulated), and 9547 DEGs (4946 up-regulated and 4601 down-regulated) were identified in the comparison between CK1 and IR1, CK2 and IR2, CK3 and IR3, CK4 and IR4, and CK5 and IR5, respectively (**Figure 3C**). The DEGs with annotations identified from five stages were listed in **Table S4-S8**. The common and unique DEGs among the five comparisons were exhibited in a Venn diagram, which showed 2153 DEGs were expressed in all the five comparisons (**Figure 3D**). Based on above analysis, the changes in a large number of transcripts between CK and IR suggested obvious difference in gene expression profile, which might be related to the difference in metabolite accumulation and resistance between CK and IR.

**Figure 4:**
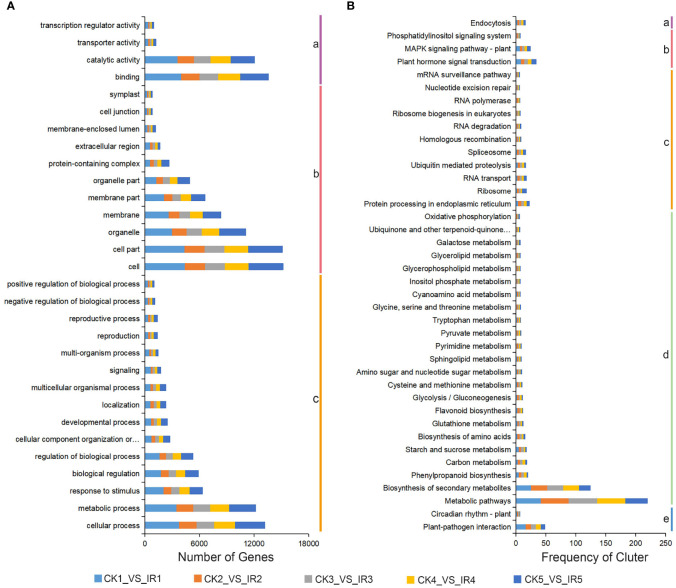
The GO classification and KEGG enrichment analysis of DEGs. **(A)** The GO classification of DEGs from five comparisons (IR1 vs. CK1, IR2 vs. CK2, IR3 vs. CK3, IR4 vs. CK4, and IR5 vs. CK5). a:molecular function, b: cellular component, and c: biological process. **(B)** The KEGG pathway enrichment of DEGs in five comparisons. a: cellular processes, b: environmental information processing, c: genetic information processing, d: metabolism, and e: organismal systems.

To explore the function classification of the above DEGs, a Gene Ontology enrichment analysis was performed. The DEGs were annotated into 54 GO terms, which were classified into three major GO categories including 16 invoving cellular component, 12 associated with molecular function, and 26 involving biological process base on five comparisons (CK1 versus IR1, CK2 versus IR2, CK3 versus IR3, CK4 versus IR4, and CK5 versus IR5) (**Table S9**). The top 30 GO terms were shown in **Figure 4A**, concerning the cellular component category, the major GO term were cell, cell part, and organelle. In molecular function, most DEGs were involved in binding, catalytic activity, and transporter activity. The major GO terms of biological process were cellular process, metabolic process, and response to stimulus. To further analyze the biological function of the above DEGs, all annotated DEGs were subjected to KEGG pathway analysis. The results revealed 135 (CK1 versus IR1), 132 (CK2 versus IR2), 132 (CK3 versus IR3), 130 (CK4 versus IR4), and 128 (CK5 versus IR5) KEGG pathways were exposed, which were divided into five categories: genetic information processing, cellular processes, metabolism, environmental information processing, and organismal systems. The details of KEGG pathways were listed in **Table S10**. The summary of top 40 common KEGG pathways in five comparisons were showed in **Figure 4B**. These results revealed that metabolic pathways was the most significantly enriched followed by biosynthesis of secondary metabolites and plant-pathogen interaction. In conclusion, DEGs analysis suggested that the metabolite change in resistant and susceptible varieties might be due to the differentially expressing of genes involved in these metabolic processes.

**Figure 5:**
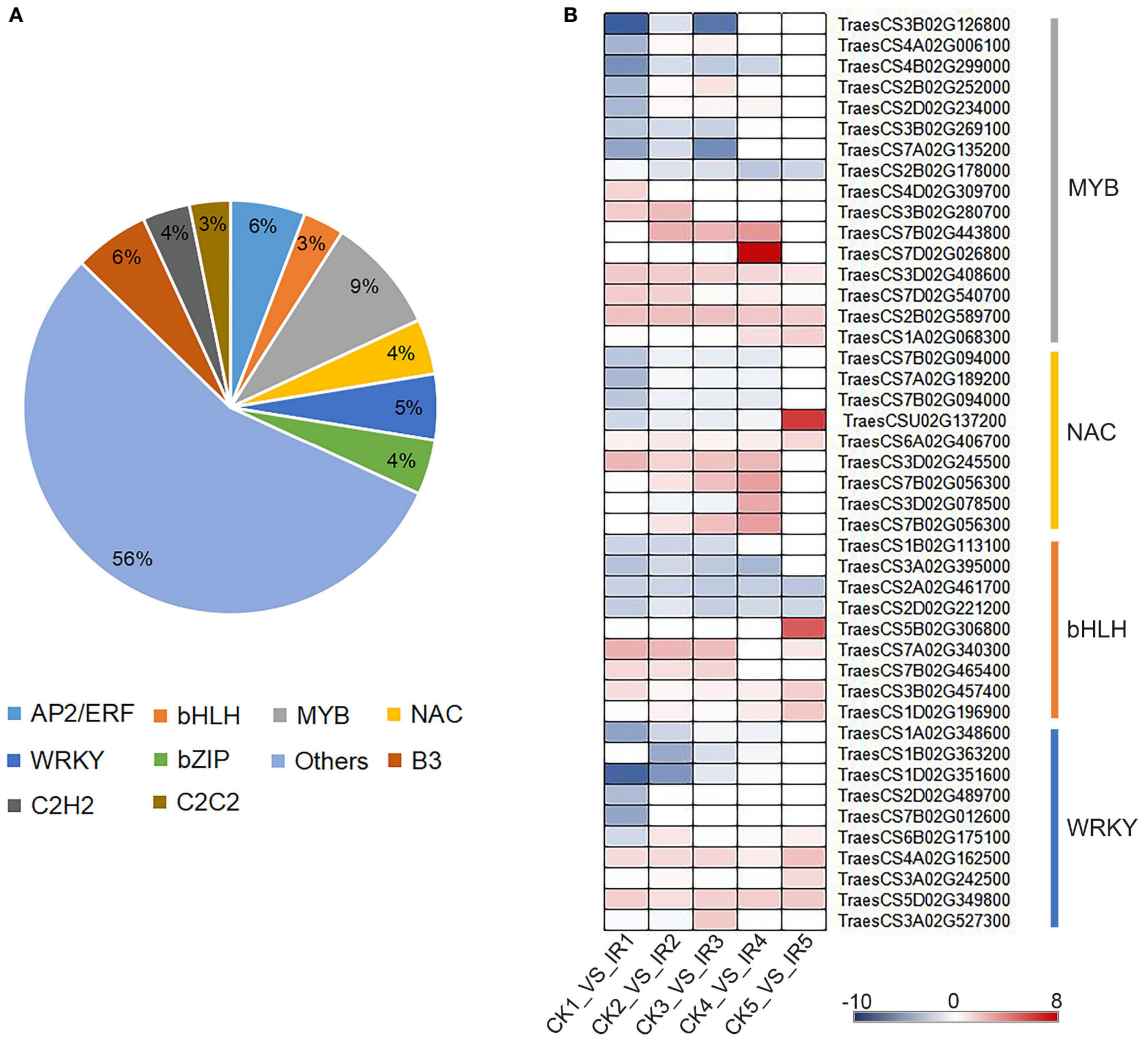
The classification and expression pattern of differentially expressed transcription factors. **(A)** The family distribution of transcription factors. **(B)** A heat map exhibit the expression pattern of transcription factors in different comparisons according to log2 fold change. The colors range from blue to red indicate low to high expression.

Considering the essential functions of transcription factors (TFs) in regulating genes expression, we also investigated their expression profiles. A total of 973 differentially expressed TFs from five paired comparisons were divided into 73 categories (**Table S11**). These TFs were distributed in 10 major families, including 57 AP2/ERF, 31 bHLH, 88 MYB/MYB-related, 50 WRKY, 42 bZIP, 539 Others, 57 B3, 36 C2H2, 42 NAC, and 31 C2C2. (**Figure 5A**). The TFs families MYB, bHLH, NAC, and WRKY play a vital role in plant defense against biotic stress. Therefore, the defense-related transcription factors against maize weevils were further identified from these four families. Based on the transcriptional levels of these candidate TFs (**Figure 5B**), the TFs although derived from the same family, showed different expression trends. Most transcription factors of MYB, NAC, and bHLH showed higher transcript levels in IR than that in CK suggesting positive regulation to wheat resistance to maize weevils, and vice versa. The expression of bHLH TFs *TraesCS5B02G306800* and NAC TFs *TraesCS6A02G406700* annotated to flavonoids biosynthesis were up-regulated in the resistant variety, suggested that these two TFs might play positive roles in wheat resistance to maize weevils by activating the biosynthesis of defense flavonoids. The results revealed that these TFs might be the key regulatory factors of downstream gene related to insect resistance, which in turn contributed to difference in CK and IR to maize weevils.

### 3.4 Co-expression network analysis of DEGs

**Figure 6:**
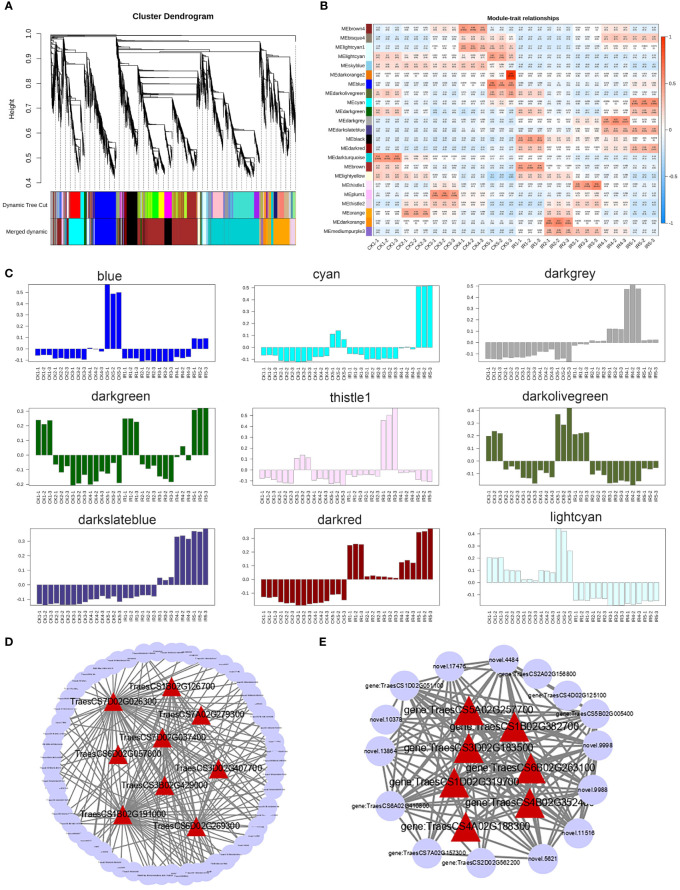
The WGCNA of differentially expressed genes. **(A)** The hierarchical clustering dendrogram of DEGs. The modules were divided base on the dynamic tree cut method. The color row was used to visualization of original modules and merged modules. **(B)** The correlation heat map between modules and samples based on Pearson's correlations. Each cell contains the corresponding correlation and p-value. The colors from green to red indicate low to high correlation. **(C)** The genes expression profiles of modules related to wheat resistance. **(D)** The genes co-expression network of blue module. **(E)** The genes co-expression network of thistle module. The hub genes in each were highlighted by red triangles.

After filtering out the genes with FPKM < 1, 22936 DEGs were used for weighted gene co-expression network analysis to identify modules and networks correlated with resistance of wheat to the maize weevils. As shown in the hierarchical cluster tree (**Figure 6A**), the co-expression modules were constructed according to the dynamic tree cut algorithm and different modules were represented by distinct colors. The genes with similar expression dynamics were divided in same modules and the minimum number of modules was set to 30 genes. After merging the modules with a correlation greater than 0.75, a total of 23 co-expression modules ranging in size from 52 genes in the thistle1 module to 5092 genes in the darkturquoise module were identified (**Table 1**). A heat map of the correlation between 23 modules and the hierarchical clustering of modules were displayed in **Figure S3**.

**Table 1:** The genes number table of each modules.

Label	Module	Gene No.	Label	Module	Gene No.
M01	blue	2195	M13	orange	2107
M02	brown4	564	M14	lightcyan	542
M03	darkgray	408	M15	darkgreen	391
M04	lightyellow	279	M16	darkolivegreen	276
M05	bisque4	126	M17	plum1	108
M06	thistle1	52	M18	darkturquoise	5092
M07	cyan	1594	M19	darkred	465
M08	skyblue	190	M20	darkslateblue	76
M09	black	2104	M21	darkorange2	84
M10	thistle2	541	M22	brown	4898
M11	darkorange	289	M23	mediumpurple3	281
M12	lightcyan1	274			

The correlation heat map between modules and different samples was shown in **Figure 6B**. And the genes expression profiles of 23 modules were shown in **Figure S4**. We mainly focused on the modules, which gene expression patterns were significantly related to wheat resistane to maize weevil at late grain filling stage. Finally, 9 special modules (blue, cyan, darkgreen, darkgrey, darkolivegreen, darkred, darkslateblue, lightcyan, and thistle1) were found and plotted the diagram of gene expression patterns (**Figure 6C**). The 465 genes in the darkred module and 542 genes in the lightcyan module displayed opposite expression pattern in susceptible and resistant samples during the whole grain filling stage. The blue module with 2195 genes and cyan module with 1594 genes were significantly correlated with stage 5 in susceptible and resistant samples, respectively. While the darkgreen module contained 391 genes and darkolivegreen module contained 276 genes with opposite expression profiles at stage 5 in susceptible and resistant samples. The 76 genes in darkslateblue, 408 genes in darkgrey, and 52 genes in thistle1 modules were positively associated with stages 3 to stage 5 in resistant samples. These results suggested the important role of these modules in the wheat resistance to maize weevils.

The hub genes, which had high connectivity in the module likely played the central role. The top 10 genes in each module were considered as hub genes according to the gene connectivity value. A total of 90 hub genes were identified from 9 special modules, which were liasted in **Table S12**. The metabolome results indicated that flavonoids biosynthesis-related pathway was the most vital for wheat defense against maize weevils. Among these 90 hub genes, *TraesCS6D02G057800* (encoding a flavonoid 3’,5’-hydroxylase) in the blue module and *TraesCS1D02G319700* (encoding a flavonol synthase) in the thistle1 module were designated to flavone and flavonol biosynthesis (ko00944) and flavonoid biosynthesis (ko00941), respectively. Thus, the subsequent analyses were performed on genes from blue and thistle modules. We analyse the KEGG pathway of DEGs in both two modules and top 18 pathways were showed in **Figure S5**. The KEGG pathway analysis indicated that the most of DEGs were enriched in plant hormone signal transduction, plant-pathogen interaction, MAPK signaling pathway - plant, flavonoid biosynthesis, and phenylpropanoid biosynthesis. Further, the hub genes of each module and high connective genes were selected and visualized by Cytoscape software to construct the interaction network. The hub genes of each module co-expression network were highlighted by red triangles (**Figures 6D, E**). These results implied that these genes might be responsible for the wheat resistance to maize weevils.

### 3.5 Integrated metabolomic and transcriptomic analysis

**Figure 7:**
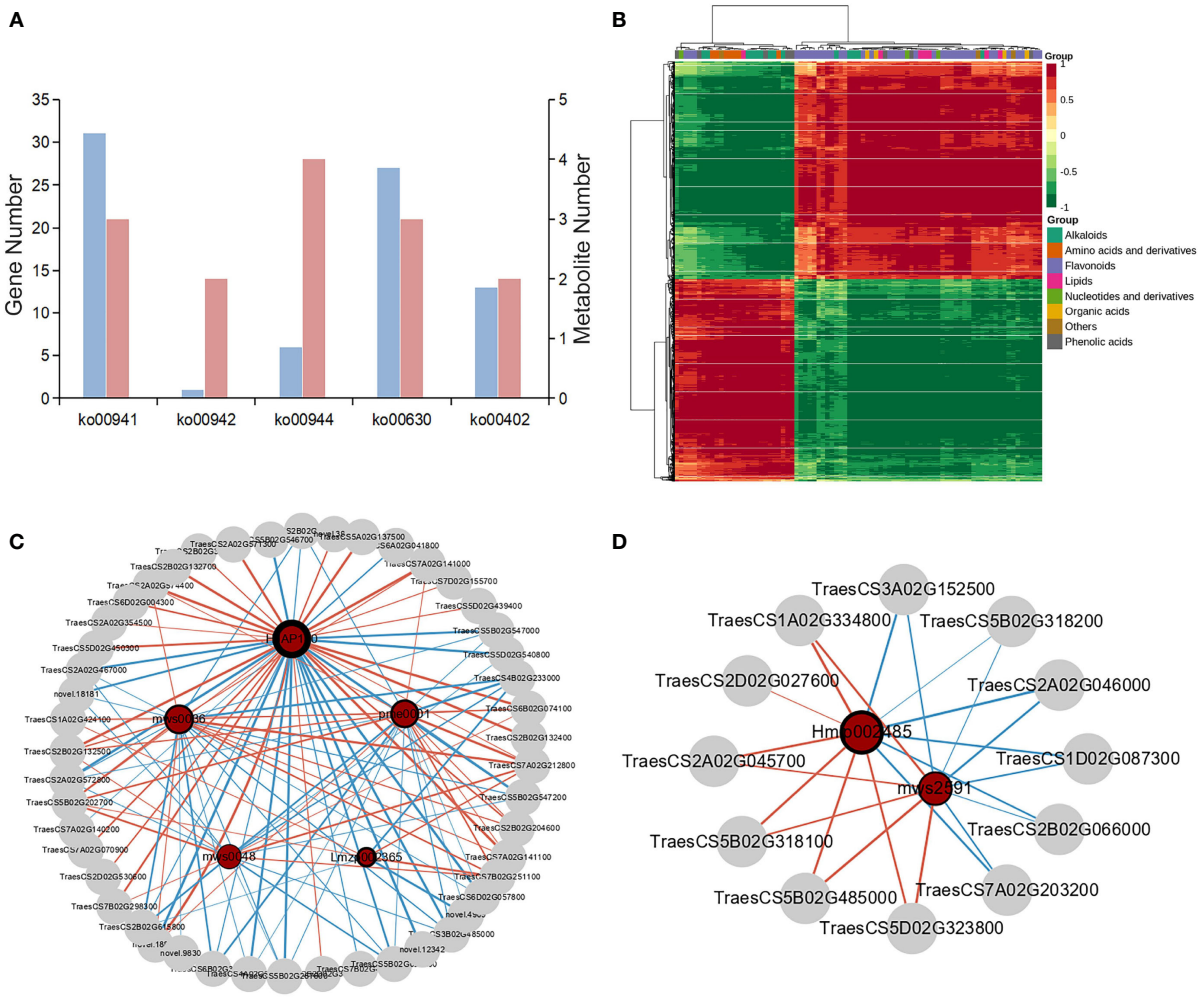
The combined analysis of trancriptome and metabolome. **(A)** The number of DAMs and DEGs enriched in common KEGG pathways. ko00941: flavonoid biosynthesis, ko000942: anthocyanin biosynthesis, ko00944: flavone and flavonol biosynthesis, ko00630: glyoxylate and dicarboxylate metabolism, and ko00402: benzoxazinoid biosynthesis. **(B)** The correlation coefficient clustering heat map between DAMs and DEGs based on Pearson's correlation coefficient. The colors from green to red indicate low to high correlation. **(C)** The correlation network diagram between DAMs and DEGs involved in flavonoids biosynthesis-related pathways. HJAP110: myricetin-O-rhamnoside, Lmzp002365: hesperetin-7-O-glucoside, mws0036: hesperidin, msw0048: vitexin, and pme0001: neohesperidin. **(D)** The correlation network diagram between DAMs and DEGs annotated in benzoxaziniod biosynthesis pathway. Hmlp002485: DIBOA-glucoside and msw2591: DIBOA. Metabolites are represented by green circles and genes are represented by gray circles. The size of the red circle represents the number of genes associated with the metabolite. The thickness of the ring frame of metabolite circles indicate the differential multiple of metabolites. The red and green lines indicate positive and negative correlation, respectively. The line thickness between nodes represents the degree of correlation between two nodes.

To explore the key genes and metabolites contributing to wheat kernels defense against maize weevils and elucidate the molecular regulatory relationships between them, the combined analysis of metabolomic and transcriptomic data was performed. First, the DEGs and DAMs were enriched to KEGG pathway and found 33 common enrichment pathways (**Table S13**). Among these pathways, 31 DEGs and 3 DAMs were involved in flavonoid biosynthesis, 6 DEGs and 4 DAMs were involved in flavone and flavonol biosynthesis, 1 DEG and 2 DAMs were associated with anthocyanin biosynthesis, 27 DEGs and 3 DAMs were enriched in glyoxylate and dicarboxylate metabolism, and 13 DEGs and 2 DAMs were associated with benzoxazinoid biosynthesis (**Figure 7A**). To clarify the relationship between DEGs and DAMs in wheat kernels defense against maize weevils, the correlation analysis was carried out. The DAMs and DEGs with Pearson′ s correlation coefficient greater than 0.8 were selected to draw the heat map which clearly exhibited strong positive and negative correlations between genes and metabolites (**Figure 7B**).

From the metabolomic and transcriptomic analysis, we concluded that flavonoids biosynthesis-related pathway plays the most important role in wheat resistance to maize weevils. The relationship between DEGs and DAMs associated with flavonoids biosynthesis metabolism was reflected by the correlation network diagram (**Figure 7B**). The differentially accumulated myricetin-O-rhamnoside was positively regulated by the genes *TraesCS7A02G141000* and *TraesCS6B02G074100* encoding flavonol synthase (FLS) and flavonoid 3′5′‐hydroxylase (F3′5′H), respectively, and negatively regulated by the genes *TraesCS7B02G412700* and *TraesCS6A02G041800* encoding dihydroflavonol 4-reductase (DFR) and anthocyanidin synthase (ANS), respectively. Hesperetin-7-O-glucoside, hesperidin, vitexin, and neohesperidin were regulated positively or negatively by the DEGs annotated in flavonoids biosynthesis-related pathway. Moreover, the correlation network of DEGs and DAMs annotated in benzoxazinoid biosynthesis and glyoxylate and dicarboxylate metabolism were also visualized in **Figure 7D** and **Figure S6**, which suggested that these genes could directly or indirectly affect the biosynthesis of corresponding metabolites.

**Figure 8:**
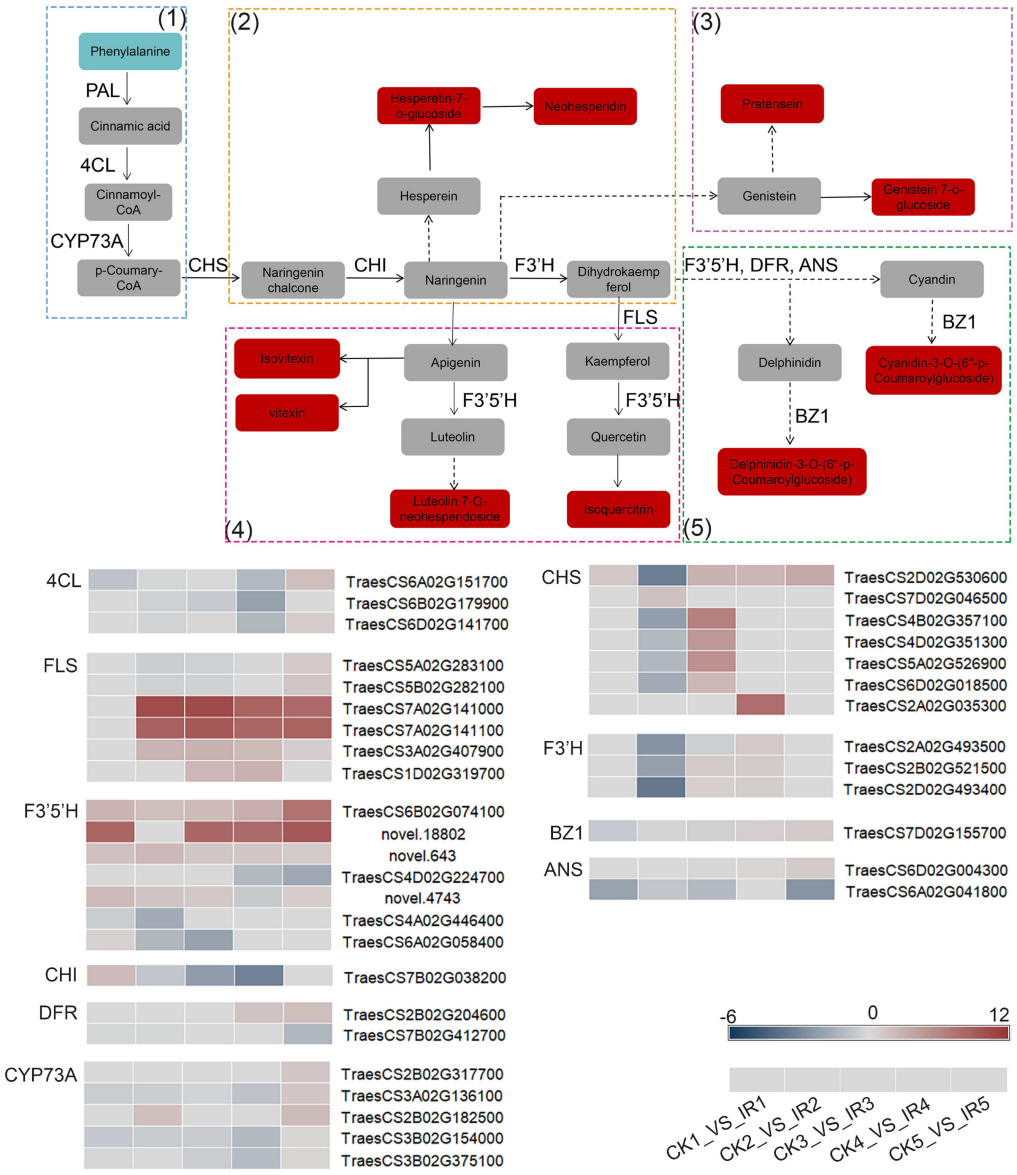
The expression profiles of DEGs and DAGs involved in flavonoids biosynthesis metabolism in the wheat kernels of resistant variety and susceptible variety. The rectangels with different colors represent the metabolites in the comparison of CK vs. IR. Red indicates up-accumulated. Sky blue indicates down-accumulated. Gray indicates not differential metabolites or not annotated metabolites. 4CL: 4-coumarate—CoA ligase; ANS: anthocyanidin synthase; CHI: chalcone isomerase; CHS: chalcone synthase; CYP73: trans-cinnamate 4-monooxygenase; FLS: flavonol synthase; F3'H: flavonoid 3'-monooxygenase; DFR: dihydroflavonol 4-reductase; F3'5'H: Flavonoid 3'5'-hydroxylase; BZ1: anthocyanidin 3-O-glucosyltransferase; PAL: phenylalanine ammonia-lyase. The expression profiles of each annotated gene encoding enzymes are presented as heatmaps. The color bar represent the value of log2 fold change with the scale ranging from blue (low) to red (high). (1): phenylpropanoid biosynthesis, (2): flavonoid biosynthesis, (3): isoflavonoid biosynthesis, (4): flavone and flavonol biosynthesis, and (5): anthocyanin biosynthesis.

In order to further understand the relationship between key genes and metabolites, the DEGs from five comparisons and DAMs concerned with flavonoids biosynthesis metabolism were mapped to the corresponding KEGG pathway diagram (**Figure 8**). The flavonoids biosynthesis metabolism was related to four pathways, including flavonoid biosynthesis, flavone and flavonol biosynthesis, isoflavonoid biosynthesis, and anthocyanin biosynthesis. As flavonoids biosynthesis was initiated from phenylpropanoid biosynthesis, the KEGG pathway map analyses of DEGs and DAMs annotated in the above five pathways were illustrated. The reactions in the phenylpropanoid pathway are catalyzed by phenylalanine ammonia lyase (PAL), and 4‐coumarate‐CoA ligase (4CL). Most of the genes encoding 4CL were up-regulated in IR. The chalcone synthase (CHS), chalcone isomerase (CHI), flavonoid 3′ -monooxygenase (F3′H), flavonol synthase (FLS), DFR, and ANS are key enzymes in the flavonoid biosynthesis, catalyzing the formation several metabolites, which flow to other branches of flavonoid biosynthesis metabolic pathway. The gene expression of *FLS*, *CHS*, *CHI*, *F3*′*H*, *DFR*, and *ANS* were increased in resistant wheat kernels. Particularly, *FLS* (*TraesCS7A02G141100* and *TraesCS7A02G141000*) and *CHS* (*TraesCS2A02G035300*) were significantly elevated in IR. F3’5’H could participate in catalytic synthesis of luteolin and quercetin which were the precursors of luteolin 7-o-neohesperidoside and isoquercitrin. The transcriptomic data showed up-regulation of *F3’5’H* (*TraesCS6B02G074100*) in five different grain filling stages. The anthocyanidin 3-O-glucosyltransferase (BZ1) participated in the formation of cyanidin-3-O-(6’’-p-Coumaroylglucoside) and delphinidin-3-O-(6’’-p-Coumaroylglucoside). The expression of *BZ1* was up-regulated in resistant variety at late grain filling stages. These results indicated that the up-regulation of these genes in IR led to the increase in the content of direct or indirect downstream flavonoids (isovitexin, vitexin, neohesperidin, and so on), which might be responsible for higher wheat resistance to maize weevils (**Figure 8**). In conclusion, we demonstrated that the resistance of wheat kernels against maize weevils was mainly related to the co-expression of these key DEGs and DAMs related to flavonoids biosynthesis pathways.

## 4 Discussion

Wheat is considered as the dominant crops and widely used for human food and livestock because of its unrivalled cultivation range and high yield (Shewry, 2009). During the wheat storage, maize weevils are the most destructive pests. The infestation of maize weevils on storage kernels caused serious losses in wheat quality and quantity (Vásquez-Castro et al., 2012; Keskin and Ozkaya, 2014). Compared with the harmful insecticides and fumigants, a safer and more effective approach to prevent maize weevil attacking was to improve the inherent defense ability of wheat (Phillips and Throne, 2010; Douglas, 2018; Kang et al., 2019). Thus, it is essential to elucidate the defense mechanism of wheat against maize weevils. In this study, a resistant variety RIL-116 and a susceptible variety RIL-72 to maize weevils were identified through screening during two consecutive growing seasons. We explored the constitutive defense mechanism of wheat against maize weevils using the combined transcriptome and metabolome data of resistant RIL-116 kernels and susceptible RIL-72 kernels. The DAMs were enriched in flavonoids biosynthesis-related pathway, benzoxazinoid biosynthesis, and glyoxylate and dicarboxylate metabolism, which were well known typical insect and pathogen defense-related metabolic pathways in previous researches. For examples, the flavonoids biosynthesis-related pathway was correlated with the resistance of wheat kernels to *Sitodiplosis mosellana*, *Zanthoxylum bungeanum* to *Fusarium zanthoxyli*, cucumber to *Sphaerotheca fuliginea*, common bean to *Tetranychus urticae*, apple leaves to *Gymnosporangium yamadai*, and alfalfa to thrips (Lu et al., 2017; Hoseinzadeh et al., 2020; Li et al., 2021; Zhang et al., 2021a; Zhang et al., 2021b; Wang et al., 2022). Consistent with our study indicated the flavonoids biosynthesis was a typical defense pathway to biotic stress, although plant-pathogen have a feeding difference compare to plant-insects. Another study revealed that the metabolic pathway of benzoxazinoids biosynthesis mediated in the direct and indirect defense of maize against *Ostrinia furnacalis* (Guo et al., 2019). The glyoxylate and dicarboxylate metabolism enhanced plant resistance to environmental stress (Zhao et al., 2020). He et al. reported that glyoxylate and dicarboxylate metabolism was significantly enriched in *Nicotiana benthamiana* response to *Chinese wheat mosaic virus* infection (He et al., 2021). Among the above pathways, flavonoids biosynthesis-related pathway was the most enriched. In addition, several DEGs and their corresponding metabolites annotated in flavonoids biosynthesis-related pathway in our study displayed differential profile between resistant and susceptible varieties. These results implied that these genes and metabolites play an essential role in the constitutive defense of wheat against maize weevils.

The secondary metabolites play important roles in plant defense against insects through direct defense (producing defense-related compounds) and indirect defense (producing volatiles to attract the natural enemies) (Aljbory and Chen, 2018; Qi et al., 2018). In particular, the flavonoids, which deter insect oviposition, feeding, and development were widely recognized as key defense substances (Monique and Simmonds, 2001; Mithofer and Boland, 2012; Dong and Lin, 2021). In the present study, flavonoids such as vitexin, isovitexin, isoquercitrin, hesperetin-7-O-glucoside, neohesperidin, myricetin-O-rhamnoside, luteolin-7-O-rutinoside, diosmetin-6-C-glucosideand accumulated in the resist variety higher than that in susceptible variety, which suggested that these flavonoids might be involved in the wheat resistance against maize weevils. In a previous study, feeding by *Sitodiplosis mosellana* induced the accumulation of hesperetin and neohesperidin in wheat (Wang et al., 2022). In *Zanthoxylum bungeanum*, the levels of vitexin and isovitexin, and luteolin-7-O-rutinoside were significantly increased after *Fusarium zanthoxyli* infestation (Li et al., 2021). The precursor of isoquercitrin was quercetin, which was reported as a resistance-related activator to improve plants resistance against pathogens and insects (Maddox et al., 2010; Golawska et al., 2014; Kang et al., 2019). Another study revealed that *Cucumis melo* synthesized diosmetin-6-C-glucosideand, which was responsible for the defense against pathogen after inoculation with F. pallidoroseum (Filho et al., 2020). Our results were consistent with previous reports further indicating that vitexin, isovitexin, isoquercitrin, diosmetin-6-C-glucosideand, and neohesperidin were the key defense compounds and the dramatic accumulation of flavonoids contributed to the constitutive defense of wheat against maize weevils. Moreover, myricetin-O-rhamnoside the most up-accumulated metabolite with the highest fold of 11.04 in resistant wheat kernels, was produced by enzymatic transformation of myricetin. Previous studies revealed myricetin was responsible for the anthelmintic effect *in vitro* (Zangueu et al., 2018). Other flavonoids such as hesperidin and isoscoparin accumulated at 3.57 fold and 1.97 fold higher levels in resistant than that in susceptible wheat kernels in our study. These results suggested that these flavonoids might play important role in the defense of wheat against maize weevils. However, the detail effect of these flavonoids substances to insects need to be further verified.

The biosynthesis of flavonoids was a complex metabolic process which derived from phenylpropanoid pathway controlled by a series of enzymes (Jiang et al., 2016; Nakayama et al., 2019). The gene expression regulated to the biosynthesis of these flavonoids were bound to change because of the content change of flavonoids substances. Consistent with the accumulation of flanovoids in resistant variety, the combined transcriptomic and metabolomic analysis in this study showed that most of the genes related to flavonoids biosynthesis, including *TraesCS7A02G141100*, *TraesCS7A02G141000*, and *TraesCS6B02G074100* were in varying degrees up-regulated in resistant variety. Our results consistent with previous reports indicating that the structural genes encoding flavonoids biosynthesis-related enzymes were involved in the defense against insects and pathogens. For examples, a flavonoids biosynthesis-related gene flavanone 3-hydroxylase (F3H) in rice was considered positively mediate the flavonoid levels and brown planthopper resistance (Dai et al., 2019). Several key genes involved in flavonoids biosynthesis were significantly induced in common bean after *Tetranychus urticae* attacking (Hoseinzadeh et al., 2020). The expression of flavonoid biosynthesis genes such as CHS, DFR, ANS, and FLS increased more than 10-fold after *Gymnosporangium yamadai* infection in apple leaves (Lu et al., 2017). Thus, our study provided a large number of candidate genes related to wheat defense against maize weevils, which serve as an important guideline for manipulating wheat defense ability against maize weevils by genetic engineering approach.

Benzoxazinoids represent an important class of defensive secondary metabolites against insects and pathogen in crops, such as such as maize, wheat and rye (Niemeyer, 2009; Stahl, 2022). Benzoxazinoids are commonly present as glucoside in plants, and are hydrolyzed to toxicity aglucones by glucosidases after insects and pathogens attacking (Niemeyer, 2009; Wouters et al., 2016). We found that the levels of benzoxazinoids DIBOA and its glucoside DIBOA-glc were decreased in the resistant variety. The first toxic material in benzoxazinoids biosynthesis pathway was DIBOA (Frey et al., 2009). The decrease of DIBOA conduced to reduce auto-toxicity in plants. A previous study reported that DIMBOA-Glc was transformed to HDMBOA-Glc by the action of O-methyltransferase in wheat and maize (Li et al., 2018). *Ostrinia furnacalis* attacking in maize induced a decrease in DIBOA-glc and DIMBOA-Glc and a significant increase in HDMBOA-Glc, which was thought entrust more resistance to insects (Guo et al., 2019). We speculated that the reduction of DIBOA-glc might lead to the accumulation of downstream major defense substance such as DIMBOA-Glc or HDMBOA-Glc. Thus, the contributions of benzoxazinoids to wheat kernels defense against maize weevils need to be further investigation.

Our study indicated that the higher accumulation of defense flavonoids contributed the most to the stronger constitutive defense ability in the resistant variety than the susceptible variety. Chemical defense-related metabolites were effective against multiple insects and pathogens, for instance, cinnamic acid plays a defensive role against *Sitodiplosis mosellana*, *Scirpophaga incertulas*, *Nilaparvata lugens* (Usha Rani and Jyothsna, 2010; Wang et al., 2022). Additionally, coumarin reduced the natality rate of *Aphis craccivora* and the survival rate of *Acyrthosiphon pisum* (Mansour et al., 1982; Kang and Wang, 2019). The constitutive defense flavonoids were always accumulated in resistant wheat kernels regardless of the presence of insects. Thus, we speculated that resistant wheat variety had wide-spectrum defense against multiple insects, not just against maize weevils. Previous study assumed that plant defense entailed metabolic cost, which reduced the resources available for growth and reproduction (Morris et al., 2006). With the limited resources, the trade-off between growth and defense in plants was particularly important. The wide-spectrum defense may reduce plant allocation costs (Koricheva et al., 2004). Therefore, we imaged the constitutive wide-spectrum defense of resistant wheat variety was not only a behavior of self-interest but also an effective way to produce resistant varieties.

In summary, comprehensive transcriptome and metabolome analyses of the constitutive defense mechanism using resistant IR and susceptible CK wheat kernels, indicated that the biosynthesis of flavonoids plays a mainly role in wheat constitutive defense against maize weevils. Different from other studies investigating induced response mechanism, this study focused on the original metabolism and transcription of host plants and elucidated the constitutive defense mechanism of wheat against maize weevils. In addition, we provided key metabolites and genes related to insect defense, which promoted the breeding of resistant varieties.

## Data availability statement

The original contributions presented in the study are publicly available. This data can be found here: NCBI, accession: PRJNA803964.

## Author contributions

XC and HL conceived the project and set the scientific objectives. LL, XG, AZ, and YL contributed to the preparation of equipment and acquisition of data. XG and LL wrote the manuscript. LL and XC reviewed and edited the manuscript. All contributed to the article and approved the submitted version.
