# Supplementary material for: Combined analysis of metabolome and transcriptome of wheat kernels reveals constitutive defense mechanism against maize weevils
Source: Front Plant Sci. 2023 May 9;14:1147145. doi: 10.3389/fpls.2023.1147145 (PMC10204651; doi:10.3389/fpls.2023.1147145)
Supplement: Supplementary file 1 [file DataSheet_1.zip › Supplementary information/Supplementary Figures.pdf]

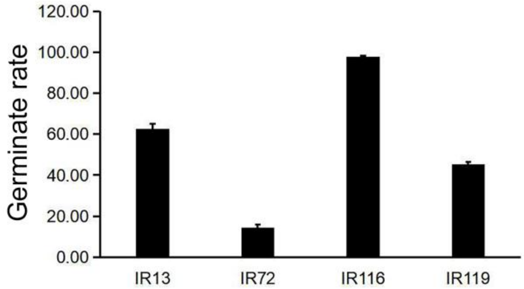

Figure S1. The germinate rate of each tested varieties after maize weevils infestation.

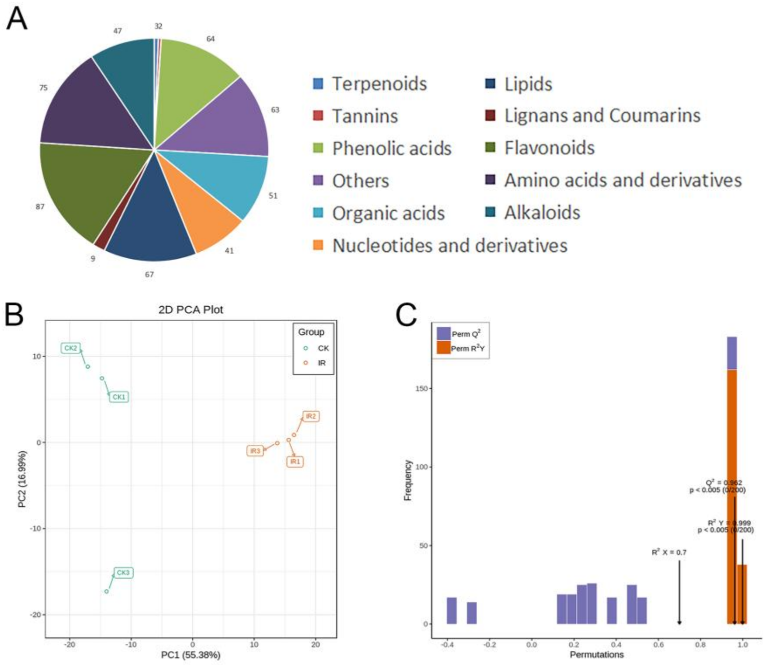

Figure S2 The metabolic analysis of RIL-116 and RIL-72.
(A) The number and classification of all identified metabolites between RIL-116 and RIL-72. (B) The principal component analysis (PCA) map of identified metabolites in RIL-116 (IR) and RIL-72 (CK). CK1-3 and IR1-3 represent three biological replicates of CK and IR, respectively. (C) The diagram of OPLS-DA model verification.

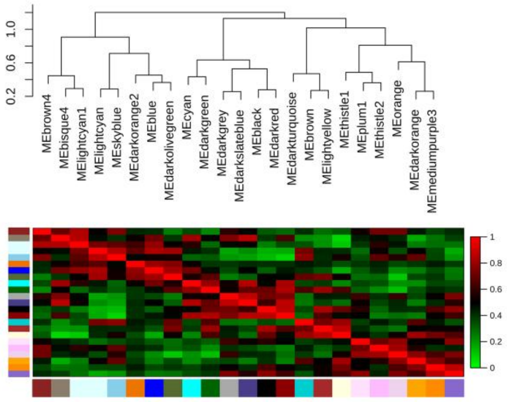

Figure S3 The heat map of modules base on eigengenes. The colors ranging from green to red represent Pearson correlation coefficients ranging from 0 to 1, indicating low to high correlations, respectively.

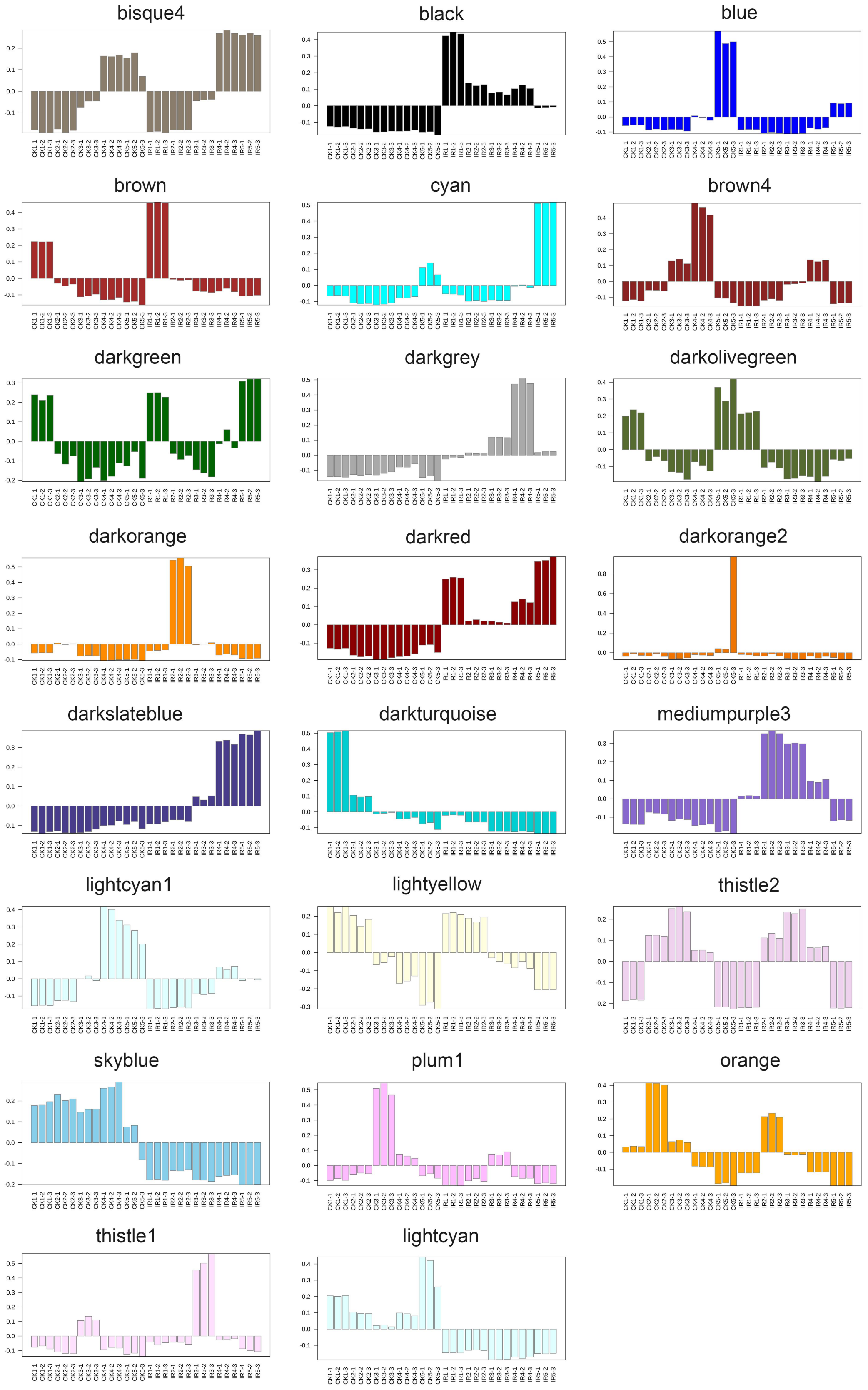

Figure S4. The genes expression profiles of 23 modules.

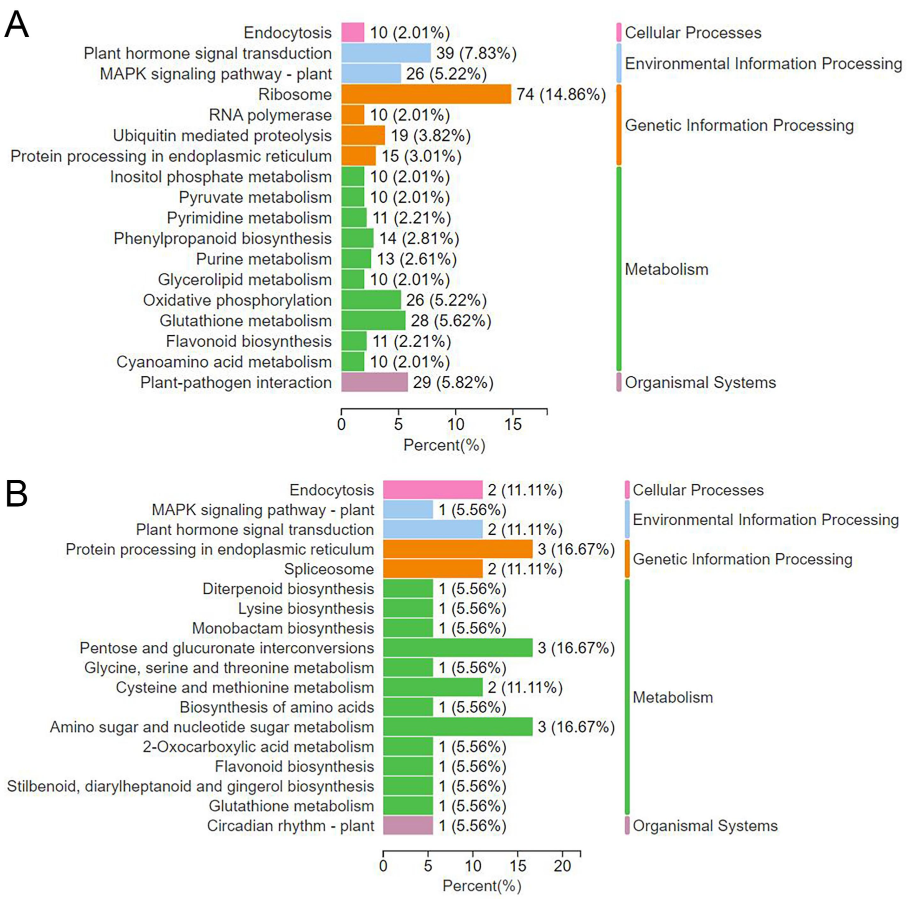

Figure S5. The KEGG pathway analysis of DEGs in blue and thistle1 modules.

(A)The KEGG pathway analysis of DEGs in blue modules. (B) The KEGG pathway analysis of DEGs in thistle1 modules.

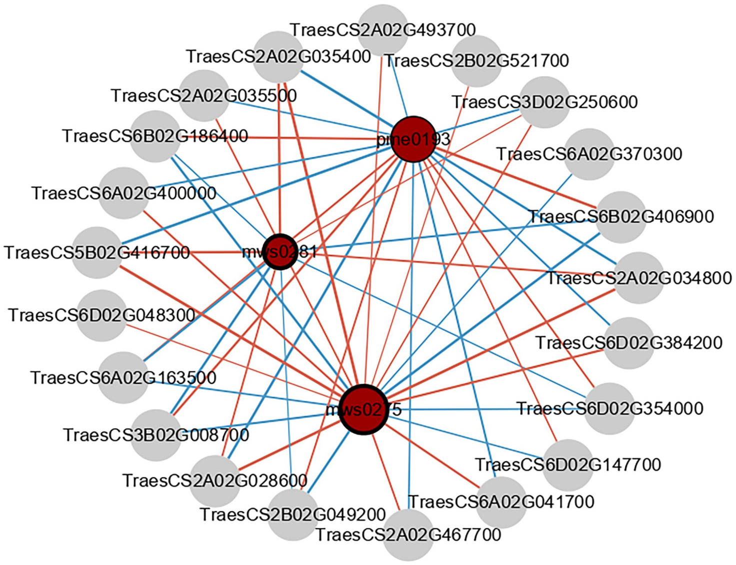

Figure S6 The correlation network diagram between DAMs and DEGs annotated in glyoxylate and dicarboxylate metabolism. Metabolites are represented by green circles and genes are represented by gray circles. The size of the red circle represents the number of genes associated with the metabolite. The thickness of the ring frame of metabolite circles indicate the differential multiple of metabolites. The red and green lines indicate positive and negative correlation, respectively. The line thickness between nodes represents the degree of correlation between two nodes. mws0275:L-(-)-Malic acid, pme0193: L-Glutamine, and mws0281: citric acid.
